# Climate drivers of large magnitude snow avalanche years in the U.S. northern Rocky Mountains

**DOI:** 10.1038/s41598-021-89547-z

**Published:** 2021-05-11

**Authors:** Erich H. Peitzsch, Gregory T. Pederson, Karl W. Birkeland, Jordy Hendrikx, Daniel B. Fagre

**Affiliations:** 1 U.S. Geological Survey Northern Rocky Mountain Science Center, 215 Mather Dr., West Glacier, MT 59936 USA; 2 Snow and Avalanche Lab, Department of Earth Sciences, Montana State University, Bozeman, MT USA; 3 U.S.D.A. Forest Service National Avalanche Center, Bozeman, MT USA

**Keywords:** Cryospheric science, Natural hazards

## Abstract

Large magnitude snow avalanches pose a hazard to humans and infrastructure worldwide. Analyzing the spatiotemporal behavior of avalanches and the contributory climate factors is important for understanding historical variability in climate-avalanche relationships as well as improving avalanche forecasting. We used established dendrochronological methods to develop a long-term (1867–2019) regional avalanche chronology for the Rocky Mountains of northwest Montana using tree-rings from 647 trees exhibiting 2134 avalanche-related growth disturbances. We then used principal component analysis and a generalized linear autoregressive moving average model to examine avalanche-climate relationships. Historically, large magnitude regional avalanche years were characterized by stormy winters with positive snowpack anomalies, with avalanche years over recent decades increasingly influenced by warmer temperatures and a shallow snowpack. The amount of snowpack across the region, represented by the first principal component, is shown to be directly related to avalanche probability. Coincident with warming and regional snowpack reductions, a decline of ~ 14% (~ 2% per decade) in overall large magnitude avalanche probability is apparent through the period 1950–2017. As continued climate warming drives further regional snowpack reductions in the study region our results suggest a decreased probability of regional large magnitude avalanche frequency associated with winters characterized by large snowpacks and a potential increase in large magnitude events driven by warming temperatures and spring precipitation.

## Introduction

Snow avalanches affect transportation corridors and settlements throughout the world. For example, avalanches impact numerous roadways and subsequently commerce in the western United States^1–5^. Understanding avalanche processes and contributory climate and weather factors aids in local and regional avalanche forecasting operations and furthers our understanding of how changing climate related avalanche activity may impact society. Although weather directly influences snowpack structure and avalanches on daily to seasonal timescales, climate works as a background influence on snowpack characteristics that can ultimately drive the avalanche climate (e.g., coastal, continental)^6–8^ or prevalent avalanche problem type^9^. In addition, variability in synoptic-scale atmospheric circulation and persistent climate modes (i.e., ocean–atmosphere teleconnections such as the El Niño Southern Oscillation (ENSO) and the Pacific Decadal Oscillation (PDO)) can have substantial effects on snowpack processes^10–16^ as well as avalanche frequency and behavior^17–20^.

Recent studies in North America investigating the association between avalanche activity and long-term climate drivers highlight a complex relationship that is challenging to disentangle from short-term synoptic-scale weather patterns. Mock and Birkeland^7^ used principal component analysis (PCA) in their investigation of atmospheric circulation and avalanche climate associations and found the Pacific-North American (PNA) teleconnections (both phases) to be correlated with several avalanche-climate patterns. Fitzharris and Schaerer^20^ associated major avalanche winters at Rogers Pass, British Columbia, Canada, with either strong zonal flow that brought heavy snow and rising temperatures, or periods of sustained meridional flow resulting in extended cold and below average snowfall followed by wet storm systems from the Pacific. In Glacier National Park (GNP), Montana, Butler^21^ suggested these same patterns produce winters with extensive avalanching using historical local media reports, National Park Service ranger logs, and local accounts. The complexity of associating climate drivers or changes in climate to subsequent effects on large magnitude avalanching warrants careful analysis and cautious inference.

The effects of climate change and increasing winter air temperature on avalanche activity trends have only recently been examined. Bellaire et al.^22^ analyzed weather, snow, and avalanche data from Glacier National Park, British Columbia, Canada, between 1965 and 2014 and found several significant trends for weather and snowpack variables consistent with a warming climate, such as warmer temperatures and less snowfall over monthly and seasonal scales. Across the European Alps, studies on climate change effects on snow avalanches have come to differing conclusions. For example, in the Swiss Alps, Laternser and Schneebeli^23^ found a gradually increasing mean snow depth, persistence of continuous snow cover, and the number of snowfall days to increase until the 1980s, after which a significant decrease in each variable was evident. Though snow conditions had changed over a 50-year period, an associated long-term change in avalanche activity was not discernable^24^, indicating these indirect measures of accumulated snowpack may not capture conditions relevant to regional avalanche generation, or that changes in climate were not yet significant enough to change regional avalanche characteristics. In the French Alps, Eckert et al.^25^ found a general decrease in dry snow avalanches since the mid-1970s as well as an increase in runout altitude (i.e., a decreased runout distance), of infrequent, large magnitude avalanches from 1980 to 2000, suggesting a potential change in avalanche character from dry snow to wet snow avalanches. Similarly, Pielmeier et al.^26^ found an increase in the proportion of wet snow avalanches in the Swiss Alps from 1952 to 2013. Eckert et al.^27^, however, found no discernible change in overall avalanche occurrence processes that could be associated with climate change over the past 60 years in the northern French Alps.

**Figure 1:**
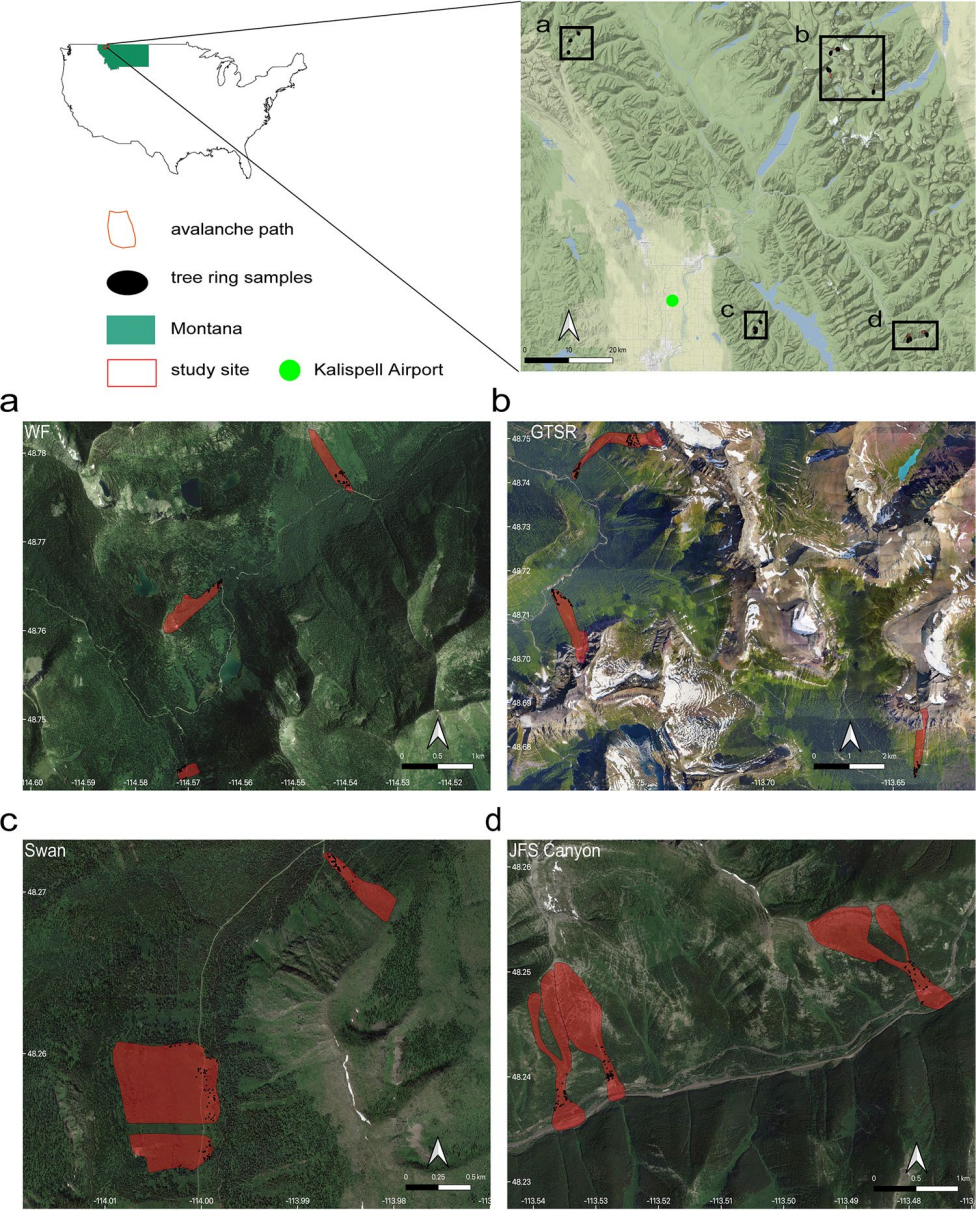
Study site. The red rectangle in the state of Montana designates the general area of the four sampling sites. The sites from upper left working clockwise are: (**a**) Red Meadow, Whitefish Range (WF), (**b**) Going-to-the-Sun Road (GTSR), central Glacier National Park (GNP), (**c**) Lost Johnny Creek, northern Swan Range (Swan), and (**d**) John F. Stevens (JFS) Canyon, southern GNP. Black dots represent sample locations. Abbreviated names of each path are in white text adjacent to red polygons (paths). Satellite and map imagery: © Google^64^ Maps produced using ggmap in R^65^.

In regions with no avalanche data or a sparse observation network, large magnitude avalanches can be inferred from dendrochronological data (i.e., snow avalanche impacted tree-ring data). This technique not only provides a record of large magnitude avalanche frequency with annual resolution, but also the ability to produce spatial reconstructions of events if sampled appropriately and strategically. Using the concept of the scale triplet^28^, we designed and sampled a regional network of 12 avalanche paths in the northern Rocky Mountains in northwest Montana, USA (Fig. 1)^29,30^. We strategically designed the sampling so that the avalanche occurrence data inherently reflects large magnitude avalanche activity on the local scale as well as the regional scale. The objective in our study is to examine broad-scale climate drivers of snow avalanches across a region with similar topography, snow characteristics, and climate influences. Therefore, the spatial extent of our samples matches the scale inherent in the explanatory climate variables we chose to examine. In addition, we define large magnitude avalanches as avalanche events characterized by low and variable frequency with a high capacity for destruction^31^. Given our sampling strategy and locations, a large magnitude avalanche would generally translate to a size three or greater on the destructive classification scale^32^. This study focuses on regional large magnitude avalanches derived from tree rings (hereafter regional avalanches).

From this regional tree-ring generated spatiotemporal avalanche dataset^30^, we address the following regional large magnitude avalanche-climate related questions: (1) are there specific seasonal climate or atmospheric circulation variables that contribute to years with common avalanche events across the region?, and (2) can we use climate-avalanche relationships to quantify and detect changing probabilities of avalanche activity through time?

## Results

### Regional avalanche chronology

To construct a regional large-magnitude avalanche chronology, we analyzed 673 total samples (614 cross sections and 59 cores) from 647 trees for growth disturbances (GD) related to avalanche damage. Within these samples we identified 2134 GDs covering a period of record spanning 1636 to 2017. The oldest tree sampled was 367 years old, and the mean age of the sampled trees was 73 years. The most common species in our dataset is *Abies lasciocarpa* (*ABLA*, sub-alpine fir) followed by *Pseudotsuga menziesii* (*PSME*, Douglas-fir) and *Picea engelmannii* (*PCEN*, Engelmann spruce). The oldest growth disturbance response dates to 1655. Using an *I*_*t*_ index that provides an indication of the number of GDs per samples alive in any given year combined with a minimum number of GDs based on sample size helped to distinguish actual avalanche signal from noise. In addition to this double threshold, a weighted index W_it_, accounts for the quality classification threshold of each GD (see Peitzsch et al.^29^ for specific equations) and results in the identification of 30 regional large-magnitude avalanche years over 1867 to 2017 (Fig. 1, Table 1).

**Table 1:** Avalanche chronology characteristics for the various sub-regions and overall region.

	WF	GTSR	Swan	JFS	Region
# of avalanche years	12	14	13	18	30
RI—median	7	8	4	3	3
RI—mean	6.27	11.35	11.25	4.94	5.21
RI—min	2	1	1	1	1
RI—max	13	53	54	16	53
1/RI	0.14	0.13	0.25	0.33	0.33
σ	3.69	13.48	15.70	4.60	9.53

RI = return interval.

### Climate conditions characterizing avalanche years

**Figure 2:**
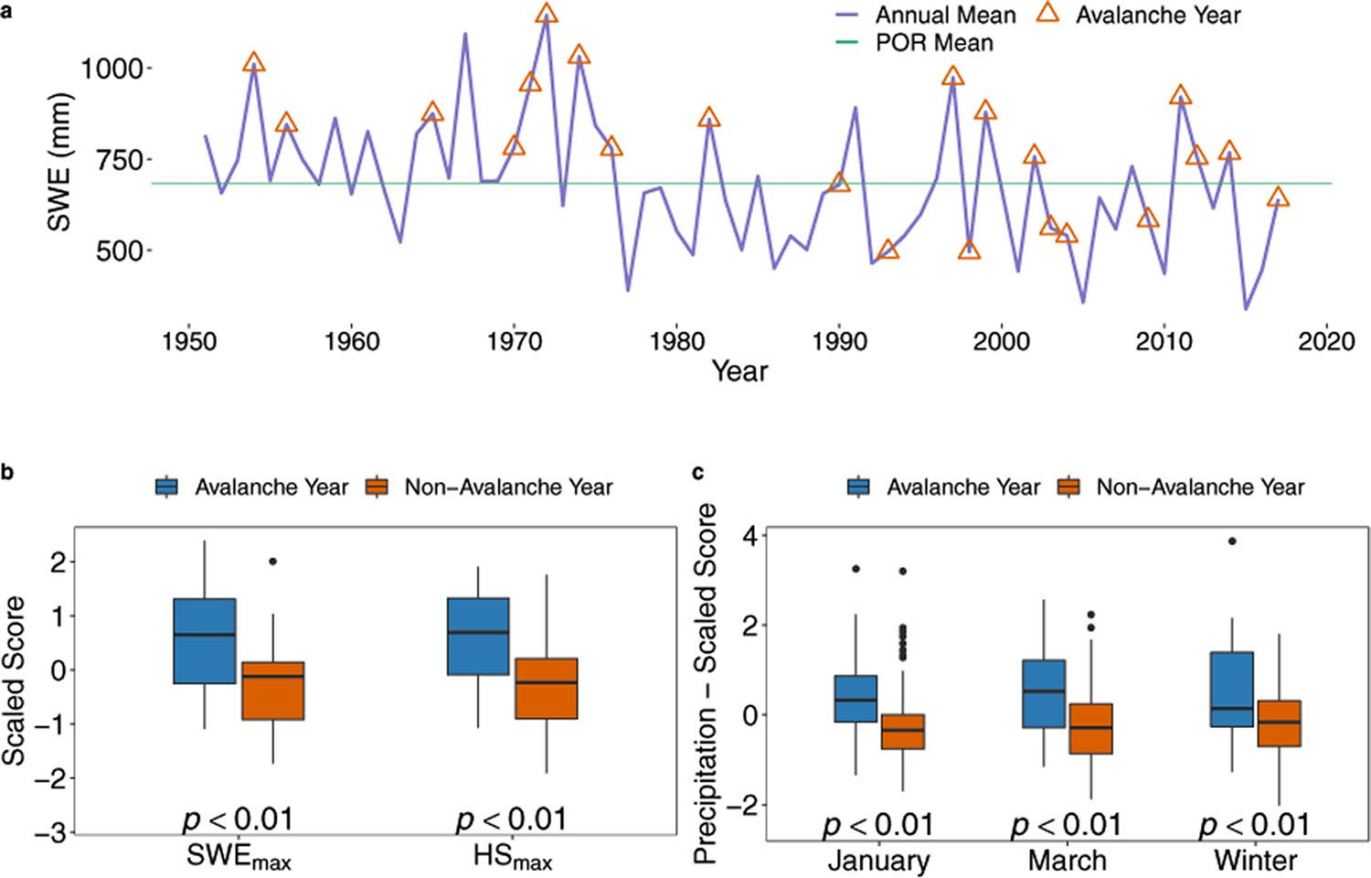
Time series and boxplots of snowpack and winter precipitation. Time Series of snow water equivalent (SWE) (**a**) with avalanche years represented by red triangles and the annual mean SWE for the period of record (green horizontal line). SWE values between avalanche (blue box) and non-avalanche (red box) years for (**b**) regional maximum snow water equivalent (SWE_max_) and maximum snow depth (HS_max_), and (**c**) significant monthly and winter precipitation totals. Horizontal black lines in the boxplots represent median values. (**a**) Displays only 23 out of 30 identified avalanche years because the analyzed period of record of SWE and HS begins in 1950 due to sparse data prior to 1950.

**Figure 3:**
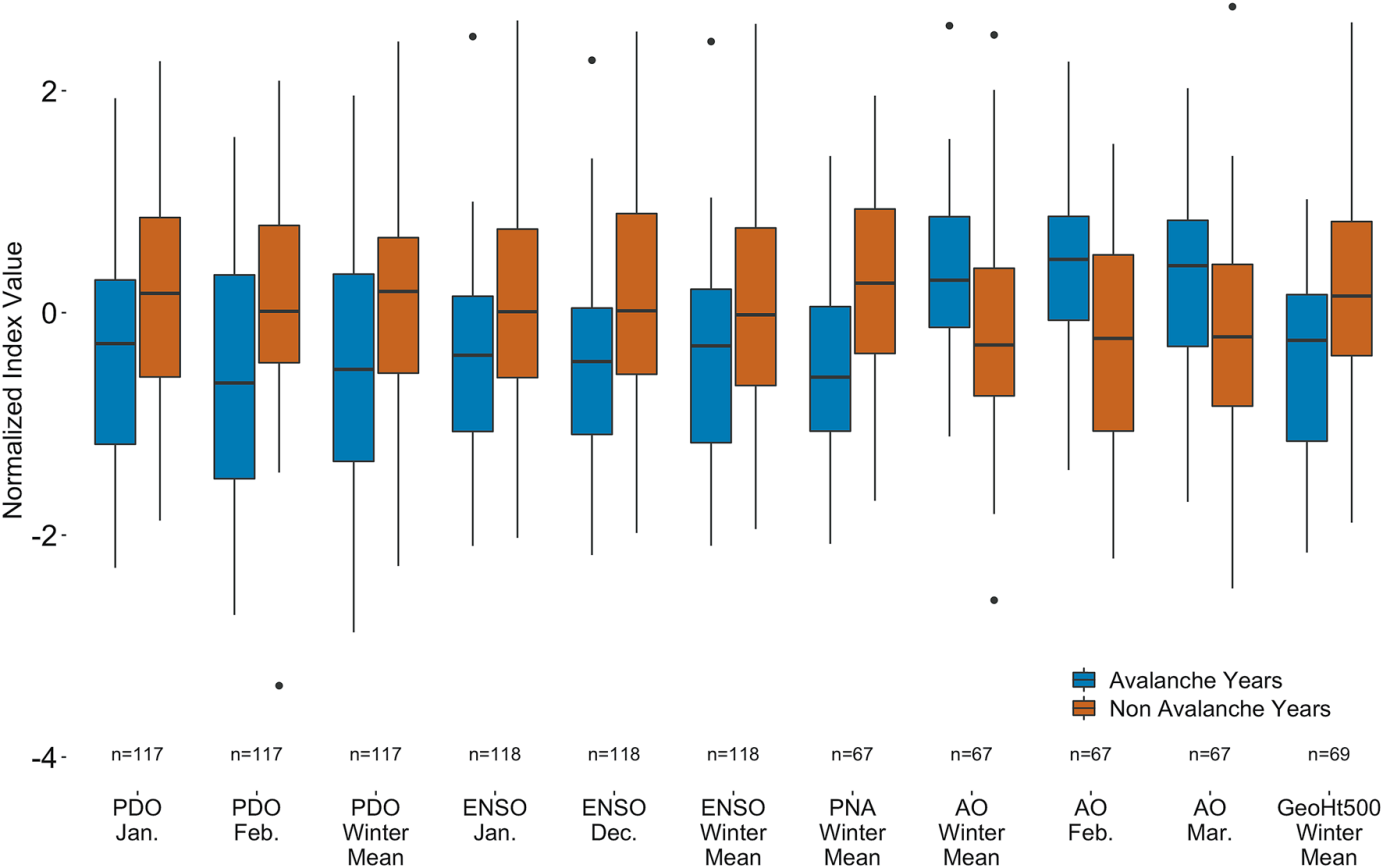
Boxplots of atmospheric variables and indices exhibiting a *significant* (*p* = 0.05) difference between conditions occurring in avalanche (blue) versus non-avalanche (red) years using the Wilcoxon rank-sum test. Sample size varies due to varying periods of record for each variable (Supplementary Table S6). Y-axis is the normalized indices of all variables for direct comparison. Full descriptions of results of each atmospheric variable are shown in Supplementary Table S2.

Using the Wilcoxson rank-sum test, we investigated whether significant differences in mean climate conditions occurred in avalanche versus non-avalanche years. Years characterized by large magnitude avalanches most often had greater mean seasonal maximum snow water equivalent (SWE_max_) and maximum snow depth (HS_max_), particularly early in the record (Fig. 2a,b). However, recent decades also show large magnitude avalanches during years of below average SWE_max_ and HS_max_. SWE_max_ and HS_max_ exhibit negative trends over the period of record (1950–2017), and winter (December through March) mean total precipitation exhibits a slight positive trend in the region (Supplementary Table S1). Significant (p < 0.05) differences in climate between avalanche and non-avalanche years exist for monthly total precipitation in January and March, as well as for annual winter precipitation totals (Fig. 2c, Supplementary Table S2). Accordingly, significant (p < 0.05) differences also exist between 11 related ocean–atmosphere indices and atmospheric circulation variables (Fig. 3, Supplementary Table S2). No significant differences in relationship to avalanche occurrence, however, are observed for the other monthly precipitation and temperature variables. General relationships reflect ocean–atmosphere teleconnections related to winters with above (below) average SWE_max_ and HS_max_ and the occurrence (lack) of regional large magnitude avalanches. More detailed investigation of climate-avalanche relationships is provided in the following sections.

### Multivariate analysis of surface and teleconnected climate conditions related to avalanche years

The surface climate variables and teleconnected indices exhibiting a significant difference between avalanche year types were used in a principal component analysis (PCA) to (1) identify dominant relationships within and between variables and (2) reduce the number of strongly co-linear potential predictors in a generalized linear modeling framework exploring climate-avalanche relationships. From 1950 to 2017, the PCA results in six principal components (PC1–PC6) with eigenvectors greater than one, capturing ~ 83% of the total variance across our climate datasets (Supplementary Figure S1). The first two principal components alone account for more than half of the variance within the dataset (~ 54%).

**Figure 4:**
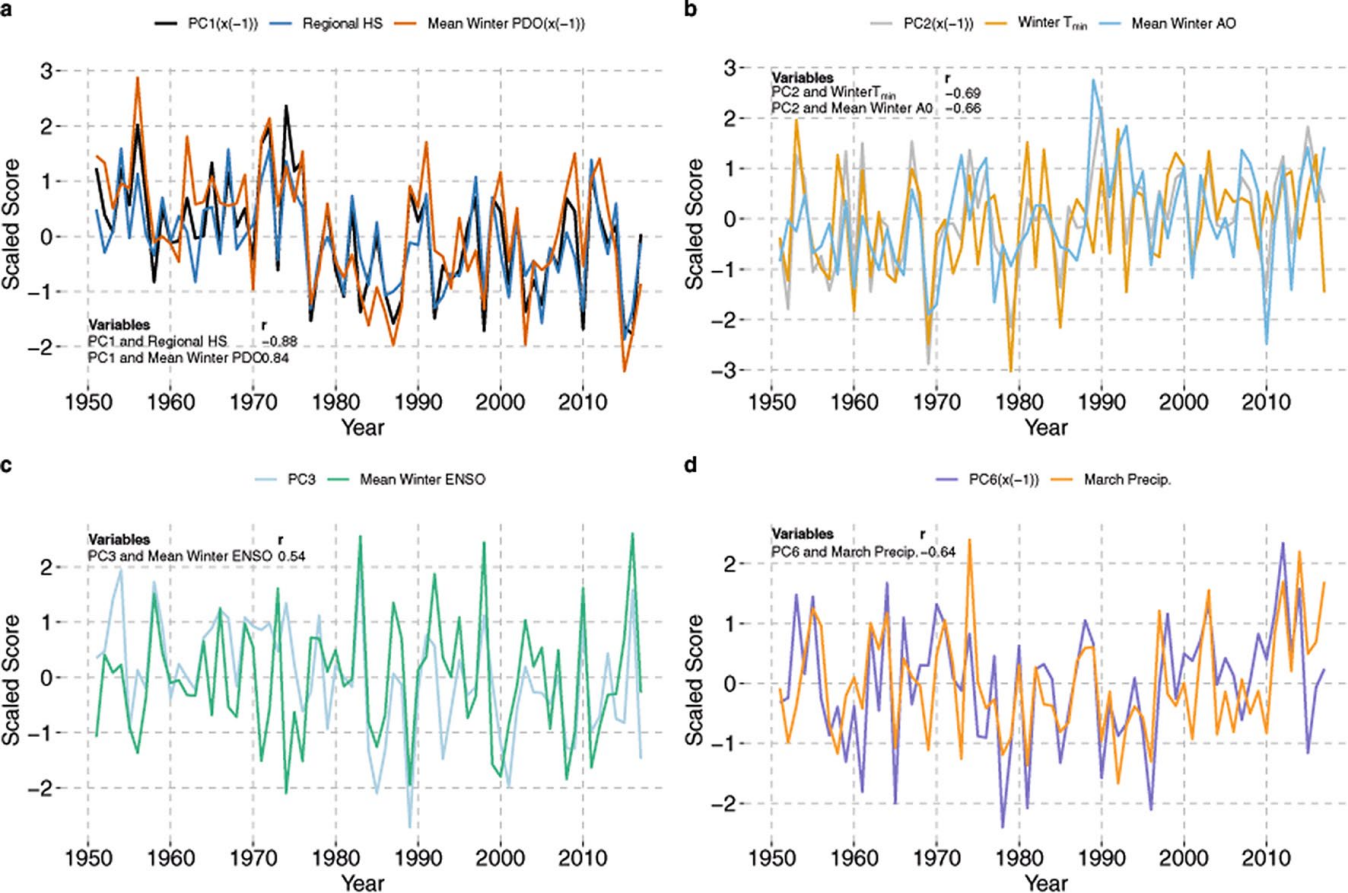
Time series plots of select principal components (PCs). (**a**) PC1 is negatively correlated with HS_max_ and positively correlated with mean winter PDO. PC1 and mean winter PDO are inverted (i.e. x(− 1)) to show decreasing trend of HS_max_. (**b**) PC2 is negatively correlated with February, March, and winter mean AO. PC2 is inverted (i.e. x(− 1)) to show increasing temperature trend. (**c**) PC3 is positively correlated with ENSO mean winter ENSO index. (**d**) PC6 is negatively correlated with March precipitation. PC6 is inverted (i.e. x(− 1)) to show correspondence with March precipitation patterns. These plots show only the greatest one or two contributors given the strong correlation of these variables to the rest of the top five contributors within each principal component (see Supplementary Figure S1).

PC1 is representative of total winter precipitation and accumulated snowpack (Fig. 4a). The largest five climate related variables contributing to PC1 (r^2^ ~ 94%) are HS_max_, February PDO, SWE_max_, PDO winter mean, and PNA winter mean (Fig. 4a and Supplementary Figure S1). Accordingly, ocean to atmosphere teleconnected indices such as the PDO and PNA are highly correlated (r = 0.74) with each other, as is the associated linkage between atmospheric circulation (i.e., the PNA) and regional HS_max_ (r = − 0.62) (Supplementary Figure S2). PC1 is negatively correlated with HS_max_ and SWE_max_ (r = − 0.88 and − 0.85, respectively) and positively correlated with February PDO, PDO winter mean, and PNA winter mean (r = 0.87, 0.84, and 0.82, respectively) (Fig. 4a and Supplementary Figure S3).

PC2 reflects winter temperatures and the atmospheric circulation indices most influential in driving year-to-year winter temperature variability (Fig. 4b). The largest five contributors (r^2^ ~ 90%) to PC2 reflect the importance of temperature in driving the variability within PC2 and include winter mean minimum temperature (T_min_), February Arctic Oscillation (AO), mean winter AO, March AO, and winter mean maximum temperature (T_max_) (Fig. 4b and Supplementary Figure S1). PC2 is negatively correlated with February, March, and winter mean AO (r = − 0.68, − 0.63, and − 0.66, respectively) as well as winter T_min_ and T_max_ (r = − 0.69 and − 0.62, respectively) (Fig. 4b).

Strong El Nino Southern Oscillation (ENSO) driven inter-annual (sub-decadal) variability in winter precipitation totals are captured and represented in PC3 (Fig. 4c). Accordingly, the largest five contributors (r^2^ ~ 83%) to PC3 include January ENSO, winter mean ENSO, December ENSO, January total precipitation, and total winter precipitation (Fig. 4c). January ENSO, winter mean ENSO, and December ENSO are positively correlated with PC3 (r = 0.56, 0.55, and 0.53, respectively). January precipitation and total winter precipitation are also positively correlated with PC3 (r = 0.5 and 0.41, respectively).

Late winter or early spring precipitation, particularly in the month of March is captured by PC6 (Fig. 4d). Though PC6 only accounts for 4.5% of the total variance in the climate dataset, this statistic simply reflects independence of individual monthly winter season precipitation totals from other climate variables, it’s later shown to be an important predictor of avalanche probability. The contribution of March precipitation to PC6 explains nearly half the variance (r^2^ ~ 41%). Other major contributors to PC6 include monthly precipitation values from November, December, January, and February (total r^2^ ~ 89%). PC6 is most strongly and negatively correlated with March precipitation (r = − 0.64) (Fig. 4d).

Finally, a subset PCA on the post-1990 dataset was run and compared against a pre-1990 subset since after 1990 many avalanche years coincide with lower SWE_max_. The subset analysis exhibited similar patterns in both explained variance as well as contributions from all PCs (Supplementary Table S3).

### Non-stationary climate drives the changing probability of avalanche years

In developing more quantitative relationships between climate conditions and large magnitude avalanche years, we fit a Generalized Linear Autoregressive Moving Average (GLARMA) model to the first six orthogonal PCs. The best-fit GLARMA model (lowest Akaike information criterion: 73.1) contains a moving average (MA) order of 6 with significant p-values from the Likelihood ratio and Wald tests (p = 0.01 and 0.01, respectively), suggesting the model is suitable. The model identifies PC1 snowpack (p < 0.01), PC2 temperature (p = 0.07), and PC6 late winter/spring precipitation (p = 0.04) as the most significant variables out of the potential six input principal components (Supplementary Table S4).

**Figure 5:**
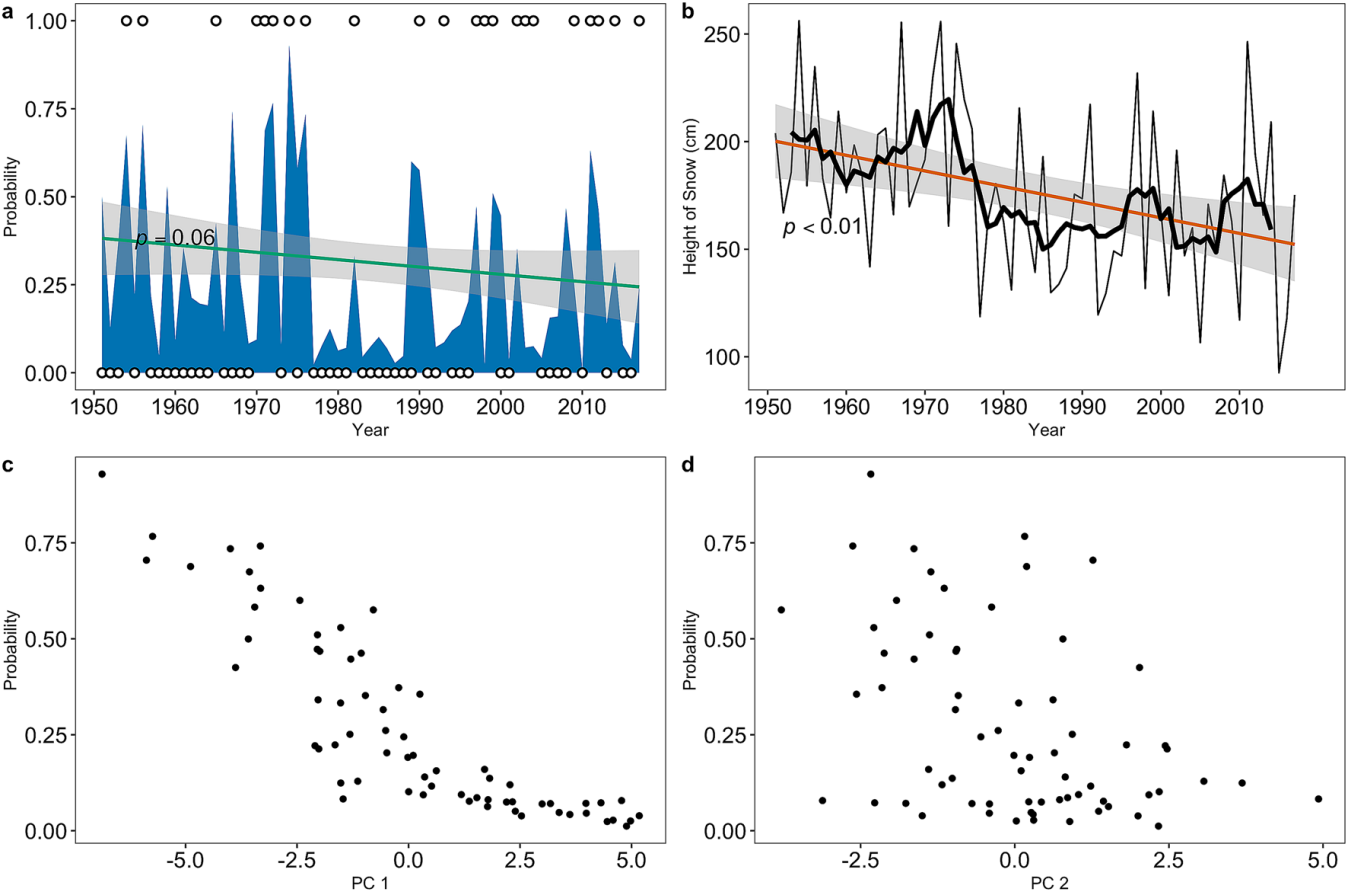
Regional avalanche probability and the major contributing variables. (**a**) Probability of an avalanche year in blue (white circles; 1 = observed avalanche year, 0 = observed non-avalanche year) using the generalized linear autoregressive moving average model. The green line represents a generalized linear model trend with 95% CIs shaded in gray. The p-value of 0.06 suggests a decreasing trend. (**b**) Time series of maximum snow depth (HS_max_) (cm) (a major contributor to and function of the first principal component (PC1)) during the period of record. The red line represents a generalized linear model trend with 95% confidence intervals shaded in gray. The p-value of < 0.01suggests a decreasing trend. The black line denotes a 6-year moving average to the time series. (**c**) The probability of an avalanche year as a function of the values of PC1. (**d**) The probability of an avalanche year as a function of the values of the second principal component (PC2).

The resulting estimates of the probability of an avalanche year through time exhibit a slight negative trend over the 1950 to 2017 time period (tau = -0.16, Sens slope = − 0.002, p = 0.06) (Fig. 5a). This roughly translates to a 2% reduction in avalanche probability per decade, and ~ 14% overall reduction over the period of record. The trend of HS_max_, the largest contributor to and function of the other atmospheric index and variable contributors of PC1, decreases over time (tau = − 0.25, Sens slope = − 0.73, p < 0.01) (Fig. 5b). Accordingly, the probability of an avalanche year decreases as PC1 become more positive (r = − 0.87) (Fig. 5c). As PC1 becomes more positive, HS_max_ and SWE_max_ both decrease, and the PDO and PNA indices become more positive. The probability of a large magnitude avalanche year and PC2 are moderately negatively correlated (r = − 0.43) (Fig. 5d), reflecting the influence of warmer winter temperatures. PC6 is weakly negatively correlated (r = − 0.35) with avalanche probability throughout the entire time series. However, we identified a positive trend (p = 0.02) trend from 1980 to 2017 in the March precipitation signal and examined large magnitude avalanche probability as a function of PC6 from 1980 to 2017. This resulted in a significant (p = 0.06) negative (decreasing) trend of PC6 through this condensed time series, which is negatively correlated with March precipitation. A slight but significant relationship exists between PC6 and the probability of large magnitude avalanche years from 1980 to 2017 (r = − 0.5, p < 0.01).

One of the expectations of a changing climate is an increase in intense or extreme precipitation events. To address this potential influence, we examined winter daily precipitation at a valley station (Kalispell, Montana (901 m)) from 1950 to 2017 (the period of record for our study). We then estimated trends using the non-parametric Mann–Kendall test for the top 1% (n = 49) and top 10% (n = 476) of winter days with positive precipitation values and found no evidence for significant trends in either daily precipitation record (Mann–Kendall, p = 0.71 and p = 0.87, respectively). We also examined winter daily snow water equivalent (SWE) accumulation at the Flattop Mountain Snow Telemetry (SNOTEL) site (1920 m) from 1970 to 2021. We calculated and tested the top 1% (n = 93) and top 10% (n = 908) of winter days with positive SWE accumulation and again found no significant trends in either daily SWE record (Mann–Kendall, p = 0.46 and p = 0.69, respectively).

Finally, to identify potential conditions unique to large magnitude regional avalanches during low snowpack conditions we examined and compared the surface climatology of years with and without regional avalanche events. Results failed to yield anything of significance related to potential climate triggers of large-magnitude avalanches occurring during recent low snowpack conditions. However, we also note the small sample size of avalanche years (n = 7) in this subset of our dataset, which is likely insufficient for statistical comparison.

## Discussion

We used avalanche damage events recorded in tree-ring samples from 12 avalanche paths to derive a regional large magnitude avalanche chronology for the U.S. northern Rocky Mountains. Our results illustrate that specific seasonal climate and atmospheric circulation variables contribute to years with common avalanche events across the region. HS_max_ and SWE_max_ exhibit negative trends throughout the period of record in our region with large magnitude avalanches occurring, on average, during years with greater HS_max_ and SWE_max_. However, over recent decades (and HS_max_ and SWE_max_ decreases), years characterized by large magnitude avalanches and negative snowpack anomalies become more frequent. This suggests that regional avalanches can and do occur during years with below average snowpack. Snow structure is an important factor in avalanche release, and with a prerequisite weak layer in the snowpack, one loading event (i.e., storm) can initiate an avalanche. If that weak layer is deep in the snowpack, even a below average snowpack can produce a large magnitude regional avalanche event. In this study region, sufficient snowpack structure data are non-existent prior to the early twenty-first century.

In our PCA and subsequent GLARMA analysis assessing climate relationships to regional avalanche activity, PC1 and PC2 explain nearly 55% of the variance of the data. The largest contributors to PC1 associated with large magnitude avalanche years are positive anomalies of HS_max_ and SWE_max_, and negative anomalies of the PDO and PNA (Fig. 4a). These contributors suggests that PC1 represents snowfall and storminess. Years with a negative PDO index, a pattern of Pacific climate variability based on sea surface temperature anomalies in the North Pacific^33^, are characterized by relatively persistent low pressure anomalies centered over western North America, which is conducive to more frequent Arctic air outbreaks. More frequent cold air outbreaks in the region provide more opportunity for faceted snow crystal growth leading to the development of weak layers in the snowpack and the preservation of snow cover later in the winter when accumulation is greatest^34^. This potential snowpack structure, combined with positive snowpack anomalies (HS_max_ and SWE_max_) associated with PC1, provides the ideal ingredients for large magnitude avalanches: deep snowpack, potential weak layer deep in the snowpack, and abundant snowfall (or at least one large storm/snow loading event). The PNA, associated with strong fluctuations in the east Asian jet stream and the jet exit region, influences precipitation in the northwestern United States. The positive phase of the PNA contributes to a decreasing fraction of snowfall in winter precipitation and an increase in storms with anomalously high freezing levels^11^, while the negative phase is associated with an intensification of a trough in the North Pacific setting the stage for increased precipitation in the western United States^7^.

Our results are consistent with earlier work that suggests the PDO and related PNA atmospheric pattern as potential drivers of snowpack change in the northern Rockies on multi-decadal scales^11^. Winters associated with positive snowpack anomalies, avalanche extremes, and increased storminess tend to be associated with negative PDO and PNA conditions in the northern U.S. Rocky Mountains^7,10,17^. The positive phase of these indices is associated with generally low snowpack, positive atmospheric height anomalies, and above-average temperatures over western Canada and the western U.S. Reardon et al.^35^ also found avalanche activity in southern GNP, Montana, documented in tree-ring records from the Shed 10-7 path (also used in this study) and historical records, correlated with positive snowpack anomalies associated with negative PDO and PNA values. This is also broadly consistent with the climate avalanche relationships shown by Butler^21^ for GNP, Montana, and Fitzharris and Schaerer^20^ in the Canadian Selkirk Mountains, located 400 km to the northwest of GNP.

Contributing ~ 14% of the explained variance in the climate dataset, PC2 represents the influence of warming temperatures on large magnitude avalanche years in our dataset. The major atmospheric contributor in PC2 to warm winter temperatures are positive anomalies in the AO (Fig. 4b). The positive phase of the AO is often associated with fewer cold air outbreaks in the mid-latitudes, while during the negative phase, the jet stream shifts toward the equator delivering more frequent outbreaks of colder air. The main avalanche years associated with PC2 are 1990, 2002, and 2003 (Supplementary Figure S3b). Avalanche events in 1990 and 2003 occurred during winters with above average temperature, and all 3 years are associated with positive February, March, and winter mean AO conditions. The contribution of temperature and the AO to PC2 within the GLARMA model suggests that several of the avalanche years, particularly in recent decades, are potentially driven by anomalous warming events. Whether this principal component is associated with wet snow avalanches is difficult to determine. We are unable to distinguish between wet and dry snow avalanches from the tree-ring record, though the more recent observational record from John F. Stevens Canyon (JFS) sub-region provides some insight. Both dry and wet slab avalanches occurred during the major March 2003 avalanche cycle in southern Glacier National Park that destroyed mature forests and closed a major transportation corridor^1^. Though warming winter temperatures represented by PC2 explain a small proportion of overall variance of our climate data, it may become an increasingly important driver of large magnitude avalanche years. For example, our results highlight that large magnitude avalanches can occur during years with below average snowpack (n = 7) (e.g., after ~ 1989), and some of those years correspond to warm temperature anomalies shown in PC2 and increasing spring precipitation. As winter and spring temperatures continue to warm in the region^36^, temperature may become a more influential driver of large magnitude avalanche years.

The results of the Wald and Likelihood Ratio tests suggest a good GLARMA model fit indicating significant relationships exist between surface climate conditions and regional large magnitude avalanche years. The model estimates avalanche probability using a 6-year moving average term and the snowpack (PC1), winter temperature (PC2), and early spring precipitation (PC6) as significant predictor variables. In related work, Ballesteros-Canovas et al.^37^ first used a GLARMA model to estimate the probability of avalanche years in the western Indian Himalayas and found increasing winter air temperature to be the sole significant predictor of avalanche years. Our results show a more nuanced and complicated relationship than increasing air temperature contributing directly to an increase in wet snow avalanche activity through time. Specifically, the significance of PC6 in the GLARMA model shows that precipitation is also an important factor, particularly spring precipitation in the month of March. At present, increasing temperatures appear to correspond with a small subset of recent avalanche years, while positive snowpack anomalies and low-pressure persistence over the region are the dominant driver of large magnitude avalanche years.

We demonstrate that it is possible to quantify non-stationary trends in climate-avalanche relationships and estimate changing probabilities of avalanche activity through time using the GLARMA model. We find a slight, but significant, decrease (~ 2% per decade, ~ 14% over the period of record) in the probability of large magnitude avalanche years from 1950 to 2017 (Fig. 5a). This result contrasts with a study using a smaller sample size of tree-ring records across a much smaller spatial extent in the western Himalaya in India that found increasing probability of avalanche activity^37^. However, our results showing decreasing avalanche probability relate to a study in the French Alps where a decrease in dry snow avalanche activity was documented over the mid-1970s to the early twenty-first century^25^. In terms of documented changes in avalanche activity, rather than avalanche probability, other regional studies in western Canada and Europe, using historical observational records, found no trend in avalanche activity^22,24,27^. These studies, however, investigated potential changes in avalanche frequency across all size classes of avalanches, not just large magnitude avalanches.

The decrease in HS_max_ and SWE_max_ through time corresponds directly to the estimated year-to-year and long-term decrease in avalanche probability. This relationship is also described by Dixon et al.^38^ who found decreased avalanche activity during El Niño years in Glacier National Park, which are typically characterized by negative snowpack anomalies. The implication of further warming driven decreases in snowpack is the likely reduction in future large magnitude avalanche activity across the region.

Annual snowpack loss exists throughout the western U.S. except for the highest elevations^39,40^. Though the probability of large magnitude avalanche years decreases through time as snowpack decreases, our results indicate large magnitude avalanches can occur during years of below average snowpack. This can partially be explained by warmer temperatures (the largest contributor to PC2) causing wet storms, or rain-on-snow events triggering large magnitude avalanches. In other words, though a decreasing probability of large magnitude avalanche years corresponds to snowpack decreases, warming temperatures can contribute to large magnitude avalanche years—at least over the near-term—through different mechanisms that are perhaps compensating for the effect of snowpack losses.

In addition, lower elevation snowpacks are more susceptible to a warming climate and exhibit greater snowpack loss^12,40,41^, but higher elevations are projected to experience stable or increasing snowpack due to increased precipitation across the region^42^. Future precipitation projections and recent observations also indicate a smaller fraction of precipitation falling as snow, particularly at lower elevations^39,43^. These projections and observations align with our results in that a slight increase in precipitation translates to snow in many of the avalanche path starting zones but less snow throughout the track and runout zone. The increased surface roughness at lower elevations due to the presence of vegetation is likely to decrease avalanche runout distances. The potential effect this increased roughness has on large magnitude avalanche occurrence is that large avalanches may still initiate at upper elevations but may not be able to reach lower elevations due to lack of snow cover in the lower track and runout zones.

Finally, we examined all avalanche and non-avalanche years associated with low snowpack anomalies for a surface climatology signal to help explain the occurrence of large magnitude avalanches during those years. The small sample size of avalanche years (n = 7) in this subset of conditions, however, limits our ability to accurately discern any significant climatic drivers potentially related to large magnitude avalanches during low snowpack conditions. Given the lack of snow structure data we are limited in our interpretation of whether the seven avalanche years can be attributed to one large storm and antecedent weak snowpack during an otherwise low snow year or to a general change in avalanche character. Given that our results suggest temperature (as part of PC2) is a significant driver of large magnitude avalanche years, it is possible that large magnitude avalanche years associated with negative snowpack anomalies are due to a changing avalanche character. In other words, there may be a shift from large magnitude avalanche years influenced primarily by storminess and abundant snowfall to large magnitude avalanche years increasingly influenced by warmer temperatures and wet snow avalanches. This possible shift coincides with other studies in the French Alps where the proportion of powder snow avalanches decreased^25^, and Swiss Alps where wet snow avalanche activity increased since the mid-1900s^26^.

Our analysis shows an increase in March precipitation from ~ 1980 to 2017. The relationship of PC6, of which March precipitation is the largest contributor, with avalanche probability from 1980 to 2017 suggests that increasing spring precipitation is a partial driver of large magnitude avalanches during years with negative snowpack anomalies. The influence of spring precipitation on the probability of a large magnitude avalanche year during the latter part of our time series combined with warming temperatures provides further evidence for a potentially increasing influence of wet snow avalanches. This potential increase in wet snow avalanches may partially explain and buffer further decreases in the probability of large magnitude avalanche years through time.

This study provided insight into the complex climate drivers of large magnitude avalanche years, but disentangling the climate signal from the more frequent and influential weather signal when analyzing avalanches remains difficult. Simply one large storm in a season combined with a snowpack with antecedent weak layers can cause a large magnitude avalanche. However, widespread large magnitude avalanching is only likely to be captured with a large enough sampling extent and intensity. Our regional sampling strategy allowed for a more scale appropriate assessment of regional avalanche activity and related climate drivers. The large spatial extent, sample size, and the nested sampling of sub-regions aligns with the synoptic influence of climate drivers.

We used a regional avalanche chronology that extends back to 1867, but high-quality observational climate record lengths required climate-avalanche analyses be conducted over 1950 to 2017. The large sample size and regional spatial extent are inherent strengths of this study, yet the non-stationarity of snowpack and temperature within a changing climate complicates the relationships between large magnitude snow avalanches and synoptic-scale climate drivers^44^. Future work with longer avalanche datasets could consider further elucidating the effect of climate drivers on avalanche frequency and avalanche character. In addition, the PCA and subsequent GLARMA model reasonably explain the historical and recent climate drivers of large magnitude avalanche years in our dataset, but the model may be limited in predicting large magnitude avalanche years in the future due to climate non-stationarity and the associated potential changes in avalanche character. Though we did not investigate changes in runout altitude of large magnitude avalanches, future work could examine the effects of low elevation snowpack loss in the region on avalanche runout across the region.

## Methods

### Regional avalanche network

The concept of the scale triplet^28^ informed how we constructed a network of three individual paths to represent a subregion, then used a network of four distinct subregions (12 paths total) to represent the annual large magnitude avalanche activity representative of the northern Rocky Mountains in northwest Montana, USA (Fig. 1)^30^. The four distinct sub-regions are located within three mountain ranges: the Whitefish Range (WF, Red Meadow Creek) and Swan Range (Swan, Lost Johnny Creek) on the Flathead National Forest, and two sub-regions within the Lewis Range in Glacier National Park (GNP) (Going-to-the-Sun Road (GTSR) and John F. Stevens Canyon (JFS)). The individual avalanche paths in each sub-region encompass a range of spatial extents from being located adjacent to one another up to locations as distant as ~ 10 km apart. The distance between any two sub-regions ranged from ~ 40 to ~ 95 km, and the total regional extent of the study area encompasses ~ 3500 km^2^ with an average per path area of 0.35 km^2^.

Northwest Montana is classified as both coastal transition and intermountain avalanche climates^7^ and exhibits characteristics of both continental and coastal climates. Avalanche paths within the study site range from ~ 1100 to 2700 m in elevation and cover all aspects except true North. The mean annual precipitation across the starting zone elevation of the avalanche paths is 1693 mm^45^, and peak snow water equivalent (SWE) typically occurs mid- to late-April with a median value of ~ 1100 mm at the highest elevation station (1920 m, 1970–2018)^46^.

Trees are susceptible to damage from geomorphic processes, such as an avalanche, and individual trees record the effects of mechanical disturbance caused by external factors. An avalanche may cause wounds on the trunk or branches. It can also locally destroy the cambium causing disruption of new cell formation. The tree then produces callus tissue, and the cambium cells overgrow the injury forming a “scar” on the tree-ring. Other types of mechanical disturbance from avalanches include reaction wood and traumatic resin ducts^47^.

Within avalanche paths we targeted an even number of samples collected along the margins of each avalanche path at varying elevations as well as trees located throughout the runout zone. We analyzed 26–109 samples per avalanche path resulting in 673 total samples from 647 suitable trees. Of those 673 samples, we collected 614 cross sections (91%) and 59 cores (9%). See Peitzsch et al.^29^ for processing methodology and tree-ring avalanche signal classification, and Peitzsch et al.^30^ for further details on cross-dating methods and accuracy calculation for this dataset.

Data preparation and all analyses were completed in *R*^48^. For tree-ring data, we used the package *slideRun*, an extension of the *burnR* library for forest fire history data^49^. We employed a multi-step process to generate a regional avalanche chronology^29^. First, we implemented a double threshold approach^50,51^ using the number of growth disturbances (GD) per year and an avalanche index *I*_*t*_^52^ to distinguish between avalanche signals and noise (i.e., irregular growth, snow creep, non-avalanche disturbance). We then used the individual avalanche path chronologies derived from the double threshold approach to calculate weighted index factor (*W*_*it*_*,* adapted from Kogelnig-Mayer et al.^53^ and Favillier et al.^51^) as a quality control measure to refine the regional and sub-regional large magnitude avalanche chronologies. We used a threshold of W_it_ = 0.2 to exclude years with low levels of confidence based on the quality of avalanche signals, as per Favillier et al.^51^, to provide us with a final avalanche chronology for four sub-regions and the overall region.

### Climate and snow data

We used historical U.S. Department of Agriculture National Resource Conservation Service (NRCS) snow course data^46^ from seven middle to upper elevation sites nearest the sub-region sites. We calculated mean seasonal maximum snow water equivalent (SWE_max_) and snow depth (HS_max_) at each of the seven sites and averaged the values to produce an annual time series of seasonal maximum snow water equivalent representative of the entire region of study (Supplementary Table S5). These snow course sites are representative of the elevations of the starting zones and tracks of the avalanche paths in this study.

Upper elevation precipitation and temperature records are only available from the early 1980s to present making them too temporally limited for use in analyses. Therefore, we used temperature records from the Global Historical Climatology Network (GHCN)^39^ to derive a longer-term (1900–2017) spatially extensive dataset^54^ for the region. To accomplish this, we applied seasonally dynamic winter (December through March) lapse rates to the Kalispell Glacier Airport site (elevation = 901 m, Fig. 1) for upper elevation sites. Then, we derived temperature lapse rates for each sub-region using the existing upper elevation network values relative to the Kalispell site. These upper elevation sites are NRCS SNOTEL sites located throughout the region. We used the gridded precipitation dataset from Daly et al.^55^ (Parameter-elevation Regressions on Independent Slope Model [PRISM]) that encompasses the study area with a 4-km resolution^45^ to estimate winter (December through March) mean total precipitation values as well as monthly values for November through March. We calculated the mean precipitation for four grids that represented each sub-region and averaged those values to produce a time series of monthly (November through March) precipitation representative of the study region.

We obtained monthly and winter mean values for atmospheric circulation and the common indices representing large-scale, ocean–atmosphere teleconnections that affect western North America climate from the United States National Oceanic and Atmospheric Administration National Centers for Environmental Information^56^ (Supplementary Table S6). We included the following variables with demonstrated effects on winter air temperature and precipitation: 500-mb geopotential height anomalies, Arctic Oscillation (AO), El Niño/Southern Oscillation (ENSO, Niño 3.4 index), Pacific Decadal Oscillation (PDO), and the Pacific-North America Index (PNA). All data underwent quality control as per Zahumensky^57^.

### Large magnitude regional avalanche climate and trend analyses

We assessed trends in the following variables: winter SWE_max_, winter HS_max_, monthly and winter temperature (maximum, mean and minimum), and monthly and winter mean precipitation using a winter season defined to be December–March. To detect trends in variables, we used the non-parametric Mann–Kendall test^58^ or the modified Mann–Kendall test^59^ if the time series exhibited serial correlation. For the trend tests and all subsequent analyses, we use 0.05 as a significance level, but do not employ a strict cutoff of 0.05^60^.

To assess potential climate drivers of regional large magnitude avalanche years, we compared all climate variables for avalanche years and non-avalanche years using the Wilcoxon rank-sum test^61^. Climate variables exhibiting a significant difference between avalanche year types were then used in a principal component analysis (PCA) along with seasonal temperature and precipitation variables to reduce dimensionality of highly correlated explanatory variables. Before PCA, all variables were scaled and centered by the mean. The number of principal components retained exhibited eigenvalues greater than one, or that the combination of the leading principal components explained at least 80% percent of the total variance^62^. We performed all analyses over a common period beginning in 1950 and extending to 2017 since this period provided maximum overlap with available climate variables. After 1990 several avalanche years coincide with lower SWE_max_, so we conducted a PCA on the post-1990 subset of the data to identify any shifts in the climate-avalanche relationship and compared that to a pre-1990 subset of the data. We then examined the relationship of these first six axes of the PCA as orthogonal predictors of a binary response (avalanche year/non-avalanche year) by fitting a GLARMA (generalized autoregressive moving average) model. This technique was used to examine avalanche activity in the western Himalaya^37^. We used the *glarma* package^63^ in *R* to specify a binomial distribution with estimation optimized using the Newton–Raphson iterative method. A likelihood ratio and Wald test allowed us to assess the goodness of fit. If the p-value was less than 0.05, we rejected the null hypothesis that the model is a generalized linear model with the same regression structure for the alternative hypothesis supporting the GLARMA model. Autoregressive (AR ($\phi ))$ and moving average (MA $(\theta )$) order values were determined from examining autocorrelation and partial autocorrelation plots. We selected the final model with specific AR and MA terms based on the lowest Akaike Information Criterion (AIC) score.

Finally, we examined the surface climatology of all avalanche and non-avalanche years associated with negative snowpack anomalies to help identify potential conditions unique to large magnitude regional avalanches during low snowpack conditions. We used a Wilcoxon rank-sum test to compare the monthly temperature and precipitation values corresponding with avalanche and non-avalanche years. We also examined trends in extreme precipitation events by using the Mann–Kendall test on the largest 1 and 10% winter daily precipitation from 1950 to 2017 at a valley station (Kalispell, Montana (901 m)) from 1950 to 2017 (the period of record for our study). We also examined winter daily snow water equivalent (SWE) accumulation at the Flattop Mountain Snow Telemetry (SNOTEL) site (1920 m) from 1970 to 2021 (the longest running mid to upper elevation site with precipitation and snow water equivalent measurements in the region).

### Disclaimer

Any use of trade, firm, or product names is for descriptive purposes only and does not imply endorsement by the U.S. Government.

## Supplementary Information

Supplementary Information.

## Data Availability

Data for this work can be found in ScienceBase repository: Peitzsch, E. H., Stahle, D. K., Fagre, D. B., Clark, A. M., Pederson, G. T., Hendrikx, J., and Birkeland, K. W.: Tree ring dataset for a regional avalanche chronology in northwest Montana, 1636-2017. U.S. Geological Survey., U.S. Geological Survey data release, https://doi.org/10.5066/P9TLHZAI, 2019.
