# Supplementary material for: Climate drivers of large magnitude snow avalanche years in the U.S. northern Rocky Mountains
Source: Sci Rep. 2021 May 11;11:10032. doi: 10.1038/s41598-021-89547-z (PMC8113570; doi:10.1038/s41598-021-89547-z)
Supplement: Supplementary file 1 — Supplementary Information. [file 41598_2021_89547_MOESM1_ESM.docx]

**Supplement**

Climate drivers of large magnitude snow avalanche years in the U.S. northern Rocky Mountains

Peitzsch, Erich H.^1,2*^, Pederson, Gregory T.^1^, Birkeland, Karl W.^3,2^, Hendrikx, Jordy^2^, and Fagre, Daniel B.^1^

^1^ U.S. Geological Survey Northern Rocky Mountain Science Center, West Glacier, Montana, USA

^2^ Snow and Avalanche Lab, Department of Earth Sciences, Montana State University, Bozeman, Montana, USA

^3^ U.S.D.A. Forest Service National Avalanche Center, Bozeman, Montana, USA

*Corresponding author: epeitzsch@usgs.gov, 215 Mather Dr., West Glacier, MT, USA 59936

Table S1: Result of Mann Kendall test for monotonic trend.

| **Variable** | ***p* - value** | $\boldsymbol{\tau}$ | **Sens slope** |
| --- | --- | --- | --- |
| T_max_ | 0.08 | 0.13 | 0.13 |
| T_min_ | 0.93 | 0.01 | 0.00 |
| SWE_max_ | <0.01 | -0.26 | -3.55 |
| HS_max_ | <0.01 | -0.25 | -0.73 |
| Precip. | <0.01 | 0.16 | 3.56 |

Table S2: Table of climate variables tested in Wilcoxon rank-sum test between avalanche and non-avalanche years. See Figures 3-4 for median values.

| **Variable** | ***p* - value** | **Variable** | ***p* - value** |
| --- | --- | --- | --- |
| PDO – January mean | 0.02 | T_min_ – Winter | 0.32 |
| PDO – February mean | 0.01 | T_min_ –January | 0.69 |
| PDO – March mean | 0.11 | T_min_ – February | 0.24 |
| PDO – December mean | 0.27 | T_min_ – March | 0.20 |
| PDO – Winter mean | 0.04 | T_min_ – November | 0.62 |
| ENSO – January mean | 0.04 | T_min_ – December | 0.27 |
| ENSO – February mean | 0.07 | T_max_ – Winter | 0.41 |
| ENSO – March mean | 0.12 | T_max_ –January | 0.36 |
| ENSO – December mean | 0.01 | T_max_ – February | 0.14 |
| ENSO – Winter mean | 0.03 | T_max_ – March | 0.13 |
| PNA – January mean | 0.19 | T_max_ – November | 0.26 |
| PNA – February mean | 0.07 | T_max_ – December | 0.51 |
| PNA – March mean | 0.14 | Precip. – January | <0.01 |
| PNA – December mean | 0.26 | Precip. – February | 0.06 |
| PNA – Winter mean | 0.03 | Precip. – March | <0.01 |
| AO – January mean | 0.76 | Precip. – November | 0.99 |
| AO – February mean | 0.03 | Precip. – December | 0.58 |
| AO – March mean | 0.05 | Precip. – Winter | <0.01 |
| AO – December mean | 0.63 | SWE_max_ – Winter | <0.01 |
| AO – Winter mean | 0.03 | HS_max_ - Winter | <0.01 |
| 500 mb Height | 0.04 |  | |

Table S3: Table of contributions (%) and r values of variables to principal components of two subsets of the full time series (1950-1990 and 1990-2017). Bold values indicate the five largest contributors in each principal component.

| **Pre-1990** |  |  |  |  |  |  |  |  |  |  |  |  |
| --- | --- | --- | --- | --- | --- | --- | --- | --- | --- | --- | --- | --- |
|  | **PC1** | **r** | **PC2** | **r** | **PC3** | **r** | **PC4** | **r** | **PC5** | **r** | **PC6** | **r** |
| **Variance Explained** | 0.40 |  | 0.15 |  | 0.11 |  | 0.08 |  | 0.06 |  | 0.05 |  |
| **500-mb height winter mean** | 6.69 | 0.57 | 5.80 | 0.18 | 2.04 | 0.05 | 1.08 | 0.02 | 3.62 | 0.05 | 0.42 | 0.00 |
| **AO Feb.** | 1.46 | 0.12 | **8.04** | **0.25** | 1.09 | 0.02 | **20.96** | **0.34** | 2.38 | 0.03 | 0.87 | 0.01 |
| **AO Mar.** | 0.30 | 0.03 | **8.19** | **0.26** | 1.17 | 0.03 | **10.26** | **0.16** | 1.19 | 0.02 | **10.20** | **0.10** |
| **AO winter mean** | 1.39 | 0.12 | **6.58** | **0.21** | 0.83 | 0.02 | **32.29** | **0.52** | 0.00 | 0.00 | 0.60 | 0.01 |
| **PNA winter mean** | 7.73 | 0.66 | 6.42 | 0.20 | 0.11 | 0.00 | 2.23 | 0.04 | 0.00 | 0.00 | 0.01 | 0.00 |
| **ENSO Jan.** | 5.46 | 0.46 | 0.63 | 0.02 | **17.99** | **0.41** | 0.87 | 0.01 | 4.19 | 0.05 | 0.40 | 0.00 |
| **ENSO Dec.** | 5.86 | 0.50 | 0.42 | 0.01 | **15.86** | **0.36** | 1.73 | 0.03 | 2.39 | 0.03 | 0.08 | 0.00 |
| **ENSO winter mean** | 5.69 | 0.48 | 0.49 | 0.02 | **17.79** | **0.41** | 1.06 | 0.02 | 2.87 | 0.04 | 0.48 | 0.00 |
| **PDO Jan** | 7.14 | 0.61 | 3.12 | 0.10 | 3.32 | 0.08 | 0.81 | 0.01 | **6.72** | **0.09** | 3.21 | 0.03 |
| **PDO Feb.** | **9.28** | **0.79** | 1.19 | 0.04 | 0.40 | 0.01 | 0.58 | 0.01 | 0.27 | 0.00 | 2.32 | 0.02 |
| **PDO winter mean** | **8.50** | 0.72 | 2.14 | 0.07 | 1.55 | 0.04 | 0.54 | 0.01 | 3.20 | 0.04 | 3.08 | 0.03 |
| **Tmin winter mean** | 0.58 | 0.05 | **21.32** | **0.68** | 3.60 | 0.08 | 1.00 | 0.02 | 7.38 | 0.10 | 3.14 | 0.03 |
| **Tmax winter mean** | 0.81 | 0.07 | **18.31** | **0.58** | 5.86 | 0.13 | 0.14 | 0.00 | **10.33** | **0.13** | **4.15** | **0.04** |
| **SWEmax** | **7.99** | **0.68** | 1.07 | 0.03 | 5.01 | 0.11 | 0.07 | 0.00 | 3.43 | 0.04 | 3.63 | 0.04 |
| **HSmax** | **8.88** | **0.75** | 0.59 | 0.02 | 2.80 | 0.06 | 0.00 | 0.00 | 4.15 | 0.05 | 2.72 | 0.03 |
| **Jan. precip.** | 4.13 | 0.35 | 3.69 | 0.12 | **9.90** | **0.23** | 0.78 | 0.01 | 3.20 | 0.04 | 0.33 | 0.00 |
| **Feb. precip.** | 3.39 | 0.29 | 2.11 | 0.07 | 0.13 | 0.00 | 3.08 | 0.05 | 4.81 | 0.06 | **16.30** | **0.16** |
| **Mar. precip.** | 2.08 | 0.18 | 1.76 | 0.06 | 0.34 | 0.01 | **7.16** | **0.11** | **10.91** | **0.14** | **37.19** | **0.37** |
| **Nov. precip.** | 2.07 | 0.18 | 3.18 | 0.10 | 1.55 | 0.04 | **9.13** | **0.15** | **8.67** | **0.11** | **6.15** | **0.06** |
| **Dec. precip.** | 2.57 | 0.22 | 1.29 | 0.04 | **5.98** | **0.14** | 0.01 | 0.00 | **20.04** | **0.26** | 3.53 | 0.04 |
| **Winter precip.** | **7.99** | **0.68** | 3.67 | 0.12 | 2.68 | 0.06 | 6.24 | 0.10 | 0.25 | 0.00 | 1.21 | 0.01 |
|  |  |  |  |  |  |  |  |  |  |  |  |  |
| **Post-1990** |  |  |  |  |  |  |  |  |  |  |  |  |
|  | **PC1** | **r** | **PC2** | **r** | **PC3** | **r** | **PC4** | **r** | **PC5** | **r** | **PC6** | **r** |
| **Variance Explained** | 0.40 |  | 0.14 |  | 0.10 |  | 0.08 |  | 0.07 |  | 0.05 |  |
| **500-mb height winter mean** | 6.11 | 0.51 | 1.67 | 0.05 | 4.49 | 0.10 | 4.04 | 0.07 | 0.10 | 0.00 | 2.87 | 0.03 |
| **AO Feb.** | 2.44 | 0.21 | **13.96** | **0.41** | 0.10 | 0.00 | 3.80 | 0.06 | 1.44 | 0.02 | 2.08 | 0.02 |
| **AO Mar.** | 0.03 | 0.00 | **15.75** | **0.47** | 1.80 | 0.04 | **15.47** | **0.26** | 2.92 | 0.04 | 2.83 | 0.03 |
| **AO winter mean** | 0.78 | 0.07 | **14.12** | **0.42** | 0.01 | 0.00 | **18.68** | **0.31** | 4.87 | 0.07 | 0.78 | 0.01 |
| **PNA winter mean** | 7.64 | 0.64 | 0.03 | 0.00 | 1.00 | 0.02 | 1.90 | 0.03 | 0.37 | 0.01 | 3.24 | 0.04 |
| **ENSO Jan.** | 7.78 | 0.65 | 2.81 | 0.08 | 0.01 | 0.00 | 2.69 | 0.05 | **10.89** | **0.15** | 0.80 | 0.01 |
| **ENSO Dec.** | **8.47** | **0.71** | 2.46 | 0.07 | 0.01 | 0.00 | 3.23 | 0.05 | 8.34 | 0.12 | 1.28 | 0.01 |
| **ENSO winter mean** | **7.91** | **0.66** | 2.91 | 0.09 | 0.02 | 0.00 | 2.29 | 0.04 | **10.17** | **0.14** | 0.92 | 0.01 |
| **PDO Jan.** | 5.52 | 0.46 | 2.29 | 0.07 | 8.99 | 0.20 | 1.25 | 0.02 | **12.17** | **0.17** | 1.13 | 0.01 |
| **PDO Feb.** | **7.43** | **0.62** | 2.77 | 0.08 | 6.35 | 0.14 | 2.15 | 0.04 | 4.78 | 0.07 | 0.57 | 0.01 |
| **PDO winter mean** | 6.68 | 0.56 | 2.87 | 0.08 | **8.51** | **0.19** | 0.87 | 0.01 | **7.43** | **0.10** | 1.49 | 0.02 |
| **Tmin winter mean** | 1.65 | 0.14 | 3.28 | 0.10 | **25.33** | **0.55** | 0.52 | 0.01 | 7.11 | 0.10 | **6.54** | **0.07** |
| **Tmax winter mean** | 2.96 | 0.25 | 1.87 | 0.06 | **21.87** | **0.48** | 0.15 | 0.00 | 7.11 | 0.10 | 2.66 | 0.03 |
| **SWEmax** | **9.33** | **0.78** | 0.57 | 0.02 | 0.02 | 0.00 | 4.91 | 0.08 | 0.14 | 0.00 | 5.38 | 0.06 |
| **HSmax** | **9.55** | **0.80** | 0.14 | 0.00 | 0.23 | 0.00 | 3.06 | 0.05 | 0.01 | 0.00 | **7.93** | **0.09** |
| **Jan. precip.** | 3.49 | 0.29 | 2.25 | 0.07 | **6.95** | **0.15** | 1.88 | 0.03 | 2.02 | 0.03 | 0.13 | 0.00 |
| **Feb. precip.** | 3.83 | 0.32 | **5.59** | **0.17** | 1.15 | 0.03 | 0.01 | 0.00 | **11.23** | **0.16** | 3.59 | 0.04 |
| **Mar. precip.** | 0.50 | 0.04 | 3.45 | 0.10 | **10.81** | **0.24** | 0.04 | 0.00 | 0.48 | 0.01 | **38.07** | **0.41** |
| **Nov. precip.** | 0.80 | 0.07 | 8.22 | 0.24 | 2.34 | 0.05 | **8.51** | **0.14** | 7.21 | 0.10 | **11.56** | **0.13** |
| **Dec. precip.** | 1.78 | 0.15 | 0.88 | 0.03 | 0.01 | 0.00 | **15.92** | **0.27** | 1.03 | 0.01 | **6.11** | **0.07** |
| **Winter precip.** | 5.32 | 0.45 | **12.10** | **0.36** | 0.00 | 0.00 | **8.63** | **0.15** | 0.16 | 0.00 | 0.03 | 0.00 |

Table S4: Best fit GLARMA model results

| **GLARMA Coefficient** | | | |  |
| --- | --- | --- | --- | --- |
|  | *Estimate* | *Std. Error* | *z-ratio* | *Pr (*$>\left\vert z \right\vert$*)* |
| $\theta=6$ | 0.96 | 0.38 | 2.55 | 0.01 |
| **Linear Model Coefficients** | | | |  |
| Intercept | -1.37 | 0.52 | -2.64 | <0.01 |
| PC1 | -0.46 | 0.14 | -3.19 | <0.01 |
| PC2 | -0.35 | 0.19 | -1.78 | 0.07 |
| PC3 | 0.05 | 0.25 | 0.20 | 0.84 |
| PC4 | -0.41 | 0.30 | 1.36 | 0.18 |
| PC5 | -0.42 | 0.27 | -1.58 | 0.11 |
| PC6 | -0.68 | 0.33 | -2.05 | 0.04 |
| **Goodness of fit tests** | | | | |
| Likelihood Test | *statistic*=6.64 | *p* = 0.01 |  | |
| Wald Test | *statistic* = 6.49 | *p =* 0.01 |  |  |

Table S5: NRCS snow course sites used in this study. Period of Record indicates the full record for each site. The analysis includes only records from 1950-2017.

| **Snow Course Site** | **Period of Record** | **Elevation (m)** | **Relevant Sub-Region** |
| --- | --- | --- | --- |
| Desert Mountain | 1937- 2017 | 1707 | Swan Range |
| Hell Roaring Divide | 1942- 2017 | 1760 | Whitefish Range |
| Marias Pass | 1934- 2017 | 1600 | JFS |
| Mount Allen No. 7 | 1922- 2017 | 1737 | GTSR |
| Ptarmigan No. 8 | 1938-2017 | 1768 | GTSR |
| Spotted Bear Mountain | 1948- 2017 | 2134 | JFS and Swan Range |
| Weasel Divide | 1937-2017 | 1661 | Whitefish Range |

Table S6: Climate, temperature, and snow property variables used in this study.

| **Climate Variable** | **Period of Record** | **Source** |
| --- | --- | --- |
| 500-mb Height Anomalies | 1948-2017 | NCDC |
| Arctic Oscillation (AO) | 1950-2017 | NCDC |
| El Niño/Southern Oscillation (ENSO, Niño 3.4 index) | 1900-2017 | NCDC |
| Pacific Decadal Oscillation (PDO) | 1900-2017 | NCDC |
| Pacific North America Index (PNA) | 1950-2017 | NCDC |
| Temperature | 1897-2017 | GHCN |
| Precipitation (mm) | 1899-2017 | PRISM |
| Snow Height (HS) | varies (Table 2) | USDA-NRCS |
| Snow Water Equivalent (SWE) | varies (Table 2) | USDA-NRCS |


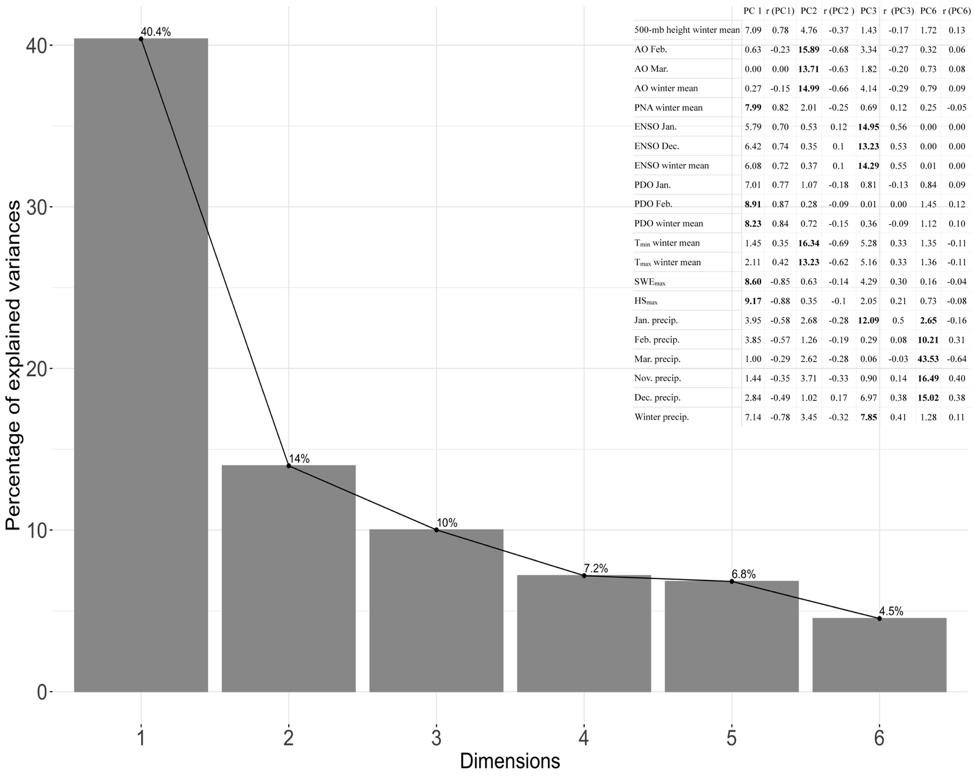


Figure S1: Scree plot of principal components (Dimensions – x-axis) and the percentage of explained variance of each (y-axis). Only the first six principal components are shown as they explain ~80% of the variance of the data. Inset table shows contributions (%) and r values of variables to principal components. Bold values indicate the five largest contributors in each principal component.


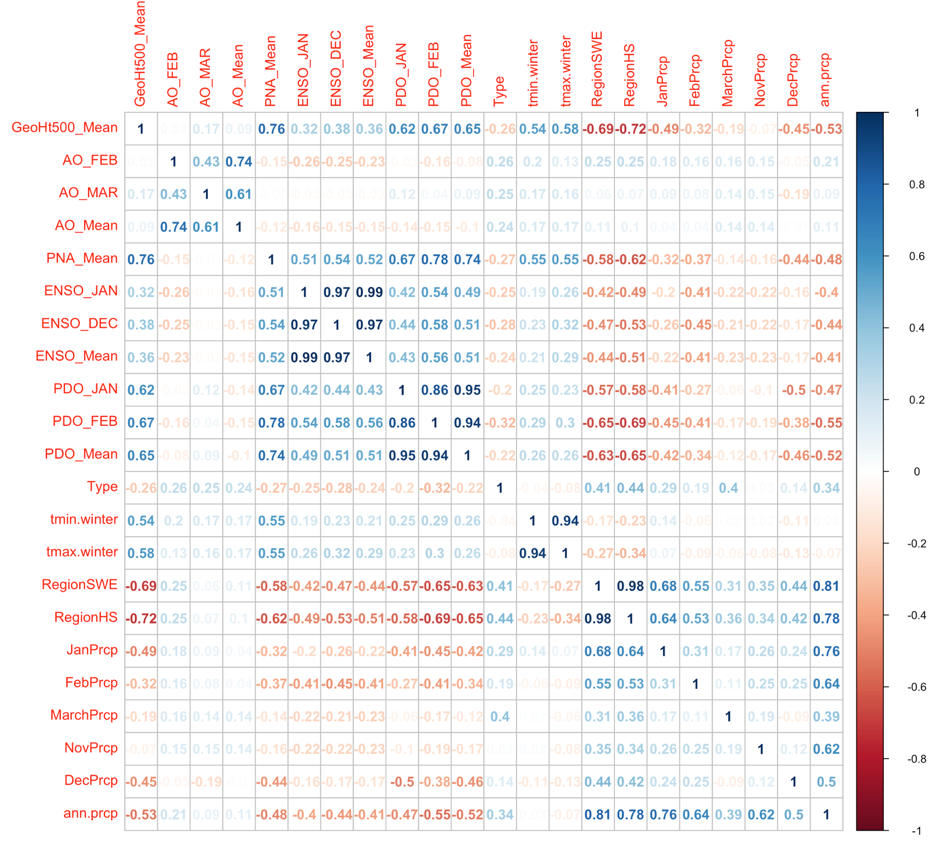


Figure S2: Correlation matrix of all variables used in the PCA.


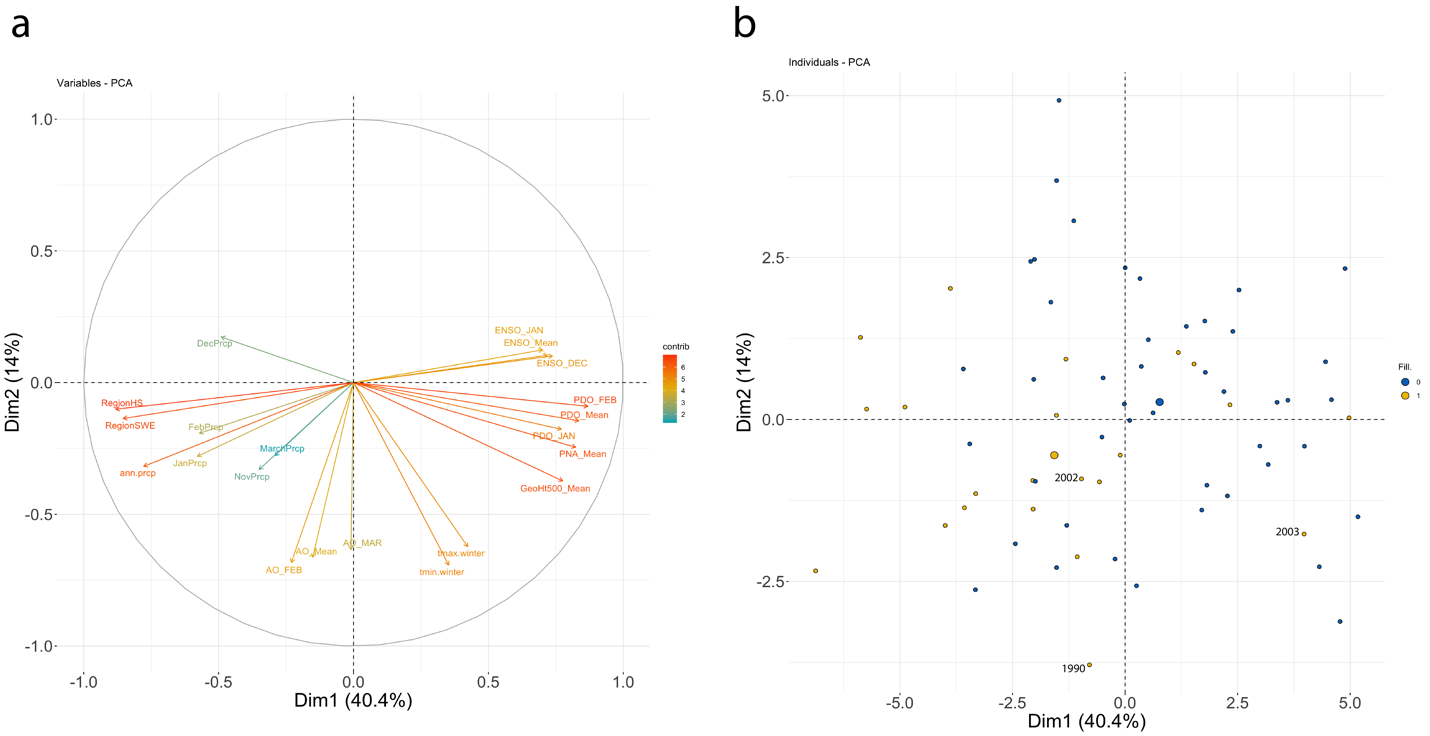


Figure S3: Plot of variables (a) in two dimensions with Dim1 (x-axis) representing the first principal component and Dim2 representing the second principal component, and plot of individual avalanche years (b) where blue points represent non-avalanche years (0) and yellow points represent avalanche years (1). The axes differ because they are scaled in the Plot A and normalized to Type (Avalanche vs. Non-Avalanche) in (b). Three avalanche years identified in (b) are annotated to the left of the associated point.
